# Additional Effects of Back-Shu Electroacupuncture and Moxibustion in Cardioprotection of Rat Ischemia-Reperfusion Injury

**DOI:** 10.1155/2015/625645

**Published:** 2016-01-11

**Authors:** Seung Min Kathy Lee, Kang Hyun Yoon, Jimin Park, Hyun Soo Kim, Jong Shin Woo, So Ra Lee, Kyung Hye Lee, Hyun-Hee Jang, Jin-Bae Kim, Woo Shik Kim, Sanghoon Lee, Weon Kim

**Affiliations:** ^1^Department of Acupuncture and Moxibustion, College of Korean Medicine, Kyung Hee University, Seoul 02453, Republic of Korea; ^2^Division of Cardiology, Department of Internal Medicine, Kyung Hee University Hospital, Kyung Hee University, Seoul 02447, Republic of Korea

## Abstract

Many preclinical studies show that electroacupuncture (EA) on PC6 and ST36 can reduce infarct size after ischemia-reperfusion (IR) injury. Yet studies to enhance the treatment effect size are limited. The purpose of this study was to explore whether EA has additional myocardial protective effects on an ischemia-reperfusion (IR) injury rat model when back-shu EA and moxibustion are added. SD rats were divided into several groups and treated with either EA only, EA + back-shu EA (B), or EA + B + moxibustion (M) for 5 consecutive days. Transthoracic echocardiography and molecular and immunohistochemical evaluations were performed. It was found that although myocardial infarct areas were significantly lower and cardiac function was also significantly preserved in the three treatment groups compared to the placebo group, there were no additional differences between the three treatment groups. In addition, HSP20 and HSP27 were expressed significantly more in the treatment groups. The results suggest that adding several treatments does not necessarily increase protection. Our study corroborates previous findings that more treatment, such as prolonging EA duration or increasing EA intensity, does not always lead to better results. Other methods of increasing treatment effect size should be explored.

## 1. Introduction

Cardiovascular disease, especially acute myocardial infarction (MI), is the leading cause of death around the world [1, 2]. Early revascularization is a critical component of decreasing its damaging effects, but reperfusion itself results in ischemia-reperfusion (IR) injury, which attenuates the overall benefits of reperfusion therapy [3].

Many studies have shown that preexposure to brief cycles of ischemia (i.e., ischemic preconditioning) and/or specific pharmacological stimuli (“pharmacologic preconditioning”) can substantially reduce infarct size after IR injury [4, 5]. Postexposure to brief cycles of ischemia (i.e., ischemic postconditioning) [6] and ischemia at peripheral sites (i.e., remote ischemic preconditioning) can also induce similar attenuation [7]. However, most of the research has focused on uncovering the mechanisms and developing pharmaceuticals while the possibility of developing other treatment methods has remained less explored.

Electroacupuncture (EA) and moxibustion are two treatment modalities that have also been shown to significantly protect rat hearts against IR injury [8, 9]. EA is usually applied for 30 min on PC6, which is located near the median nerve. Stimulation of this point can either precondition or postcondition the heart, depending on the treatment period [8, 10]. Moxibustion, on the other hand, is usually given in the form of local somatic thermal stimulation (LSTS) on PC6, and it is believed to work by increasing the production of protective proteins such as heat shock protein 70 (HSP70) and by activating heat-sensitive neural release of nitric oxide [9]. Other than EA and moxibustion on PC6, acupuncture texts also mention back-shu points, which are a set of special points located about 3 cm apart from the midline of the spine. Each set of points are designated to specific organs, and among them BL14 and BL15 have been frequently cited for use in the treatment of cardiovascular diseases [11]. However, the protective effects of these points on IR injury have not been well investigated.

Although EA, moxibustion, and BL14, and BL15 are all postulated to protect against IR injury, the physiological mechanisms in action are thought to be different. While PC6 and ST36 exert their effects through the central nervous system [12], back-shu points act directly on the dorsal ganglia and spinal nerves that provide sympathetic outflow to the heart [13]. To the best of our knowledge, no previous studies have looked into the effects of all the three combined. Some trials have attempted to enhance the myocardial protective effects of EA or moxibustion, but most of these only added or manipulated stimulation methods. Some used different acupuncture points [14], others prolonged the time of stimulation [15], and one explored moxibustion treatment using different temperatures [16], but none explored added effects.

Therefore, in this trial, we hypothesized that, in order to increase effects, treatment modalities must be appropriately and synergistically combined. As a preliminary study, we aimed to explore the possibility of increasing myocardial protective effects by successively combining standard EA, back-shu EA, and moxibustion in a rat model of IR injury.

## 2. Materials and Methods

### 2.1. Study Protocol

**Figure 1:**
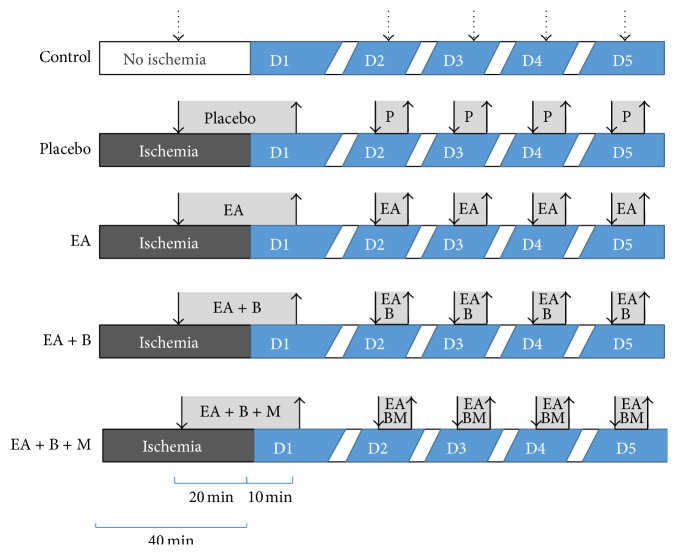
Study protocol. The control group received 30 min of anesthesia every day and no other treatment. The placebo group received acupuncture on nonacupuncture points for 20 min at the point of IR injury. The EA group received electroacupuncture on PC5, PC6, ST36, and ST37 for 20 min at the point of IR injury. The EA + B group received electroacupuncture like the EA group but was also treated with electroacupuncture on BL14 and BL15 for 20 min at the point of IR injury. The EA + B + M group received electroacupuncture like the EA + B group but was also treated with moxibustion on PC6 for 15 min for 20 min at the point of IR injury. All treatments were performed every day, and treatment was purposefully designed to overlap with time of reperfusion on the first day (D1). D, day; ischemia, ischemia induced by ligation.

Thirty-five Sprague-Dawley rats (8- to 9-week-old males, body weight: 250–300 g, Samtaco Inc., Osan, Korea) were used after a 1-week acclimation period under standard laboratory conditions. They were housed in chip-bedded cages at room temperature 24 ± 1°C and room humidity (63 ± 5%) in a 12-hour light/dark cycle. Rats were permitted free access to water and standard rat chow. On the day of the experiment, the animals were divided into five groups: (1) the control group (*n* = 7), which received no treatment except 30 min of anesthesia for five consecutive days; (2) the IR + placebo group (*n* = 7), which received 30 min of acupuncture treatment after anesthesia on nonacupuncture points to a shallow depth (5 ± 1 mm) without any electrical stimulation; (3) the IR + EA group (EA) (*n* = 7), which received EA on the left PC5, PC6, ST36, and ST37 with a frequency of 2 Hz for 30 min once daily for 5 days; (4) the IR + EA + back-shu EA group (EA + B) (*n* = 7), which received EA on the left PC5, PC6, ST36, and ST37 and on bilateral BL14 and BL15 with a frequency of 2 Hz for 30 min once daily for 5 days, and (5) the IR + EA + B + moxibustion group (EA + B + M) (*n* = 7), which received EA on the left PC5, PC6, ST36, and ST37 and on bilateral BL14 and BL15 with a frequency of 2 Hz for 30 min, along with moxibustion on left PC6 for 20 ± 5 min (Figure 1).

### 2.2. Rat IR Injury

All experimental procedures were approved by the Kyung Hee University Hospital Animal Experimentation Committee. The rats were anesthetized via intraperitoneal injection with ketamine (75 mg/kg) and xylazine (2 mg/kg). The neck and chest were shaved. Procedures were performed under endotracheal intubation with mechanical ventilation (Harvard Apparatus, MA, USA). The chest was opened through left intercostal thoracotomy, and the heart was exposed by removing the pericardium. Myocardial ischemia was produced by exteriorizing the heart with a left thoracic incision, followed by placement of a slipknot (5–0 silk) around the left anterior descending coronary artery (LAD). After 40 min of ischemia, the ligature was released and blood flow was restored.

### 2.3. Electroacupuncture

Acupuncture points for EA were selected through literature research. Many studies utilized bilateral PC6 for stimulation, but ST36 was also frequently used in conjunction with PC6 to increase myocardial protective effects [17]. Among the back-shu points, BL14 and BL15 were the most frequently mentioned acupuncture points for treatment of cardiovascular diseases [11].

**Figure 2:**
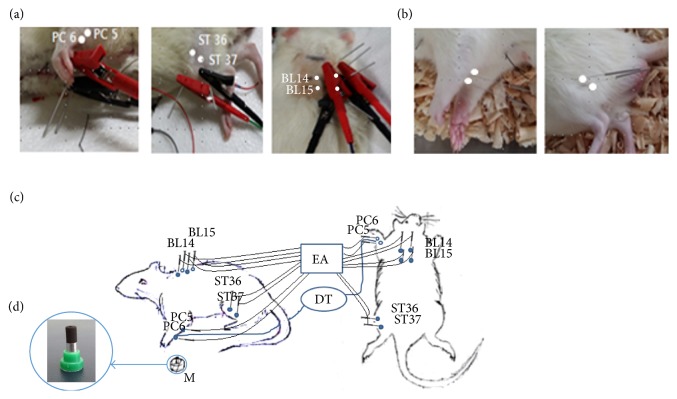
Schematic diagram of treatment and acupuncture points. (a) PC5, PC6, ST35, ST36, BL14, and BL15. To needle BL14 and BL15, the back was shaved and the location of the T4/T5 thoracic spine was estimated by palpation of significant anatomical structures. (b) Placebo acupuncture points were located away from any acupuncture points or meridians. (c) Schematic diagram showing electroacupuncture (EA) and moxibustion (M) treatment of the Sprague-Dawley (SD) rats. The temperature was continuously monitored using a digital thermometer (Giltron GT309, Seoul, Korea). (d) For moxibustion treatment, indirect moxibustion (M) was applied on the left PC6.

All acupuncture needles (0.20 × 30 mm, Dongbang Acupuncture Inc., Gyeonggi-Do, Korea) were inserted to a depth of 5 mm at PC5 and PC6 and to a depth of 1 cm at ST36, ST37, BL14, and BL15. EA was performed with an electric stimulator (ES-160, ITO, Tokyo, Japan). The motor threshold responses of the rat paws and legs were verified by visible muscle twitches of the stimulated area [18, 19]. To make sure the needles were positioned at the same points every day, each area was shaved and marked with a black tip marker. The details of acupuncture points and treatment methods are reported following the Standards for Reporting in Animal Studies of Acupuncture (STRASA) guidelines [20] (Table 1 and Figure 2).

On the day of the surgery, electric clips were connected to the acupuncture points 20 min after ischemia was induced and the snare was loosened 10 min before electrical stimulation was due to end. Additional anesthesia was administered intramuscularly if needed, but to a minimal degree. For the next four days, EA stimulation was given with a frequency of 2 Hz for 30 min under anesthesia.

### 2.4. Moxibustion

For moxibustion treatment, an indirect moxibustion cone (Ucare Int., Gyeonggi-Do, Korea) was positioned approximately ~5 cm under the left PC6 and its temperature was maintained between 38 and 45°C with a digital thermometer (Giltron GT309, Seoul, Korea).

### 2.5. Transthoracic Echocardiography

To evaluate LV function, rats were intraperitoneally anesthetized with ketamine (75 mg/kg) and xylazine (10 mg/kg) on day 5. The chest was shaved for two-dimensional transthoracic echocardiography (Vivid Q; GE Medical Systems, Milwaukee, WI, USA) with a 12 MHz probe. M-mode echocardiography of the LV was performed at the papillary muscle level, guided by two-dimensional short-axis images. LV cavity size was measured during at least three beats in each projection and averaged. The M-mode images yielded systolic and diastolic wall thicknesses (anterior and posterior) and LV end-systolic and end-diastolic diameters. LV fractional shortening was calculated as (LVEDD minus LVESD)/LVEDD *∗* 100. The ejection fraction was calculated using the Teichholz formula. The following parameters were obtained: left ventricular ejection fraction (LVEF), left ventricular fractional shortening (LVFS), left ventricular internal diameter in diastole (LVIDd), left ventricular internal diameter in systole (LVIDs), LV end-diastolic volume (LVEDV), regional wall thickness, and LV mass.

### 2.6. Infarct Size and Histological Examination

At day 5, KCl (0.6 mL) was administered intravenously to euthanize the rat. Immediately after euthanasia, the heart was removed and the area at risk was determined by negative staining. Evans blue (0.25%) was administered via the jugular vein to stain the nonoccluded area of the left ventricle (LV). The heart was excised, and the right ventricle and connective tissue were removed, leaving the LV intact. The heart was then frozen at 20°C. The frozen ventricles were sliced transversely from apex to base in 3 mm slices. Slices were incubated in 1% 2,3,4-triphenyltetrazolium chloride (TTC) buffer at pH 7.4 for 20 min at 37°C, washed in flowing water for 30 min, and immersed in 10% formalin. The TTC stained the noninfarcted regions brick red, while the infarcted myocardium remained pale. The infarct area (TTC negative) and area at risk (TTC stained) were measured with the 2000 Visual Image Analysis System, and infarct severity was calculated as a percentage of the infarct size/area at risk (IS/AAR%). The primary end point of the study was myocardial infarct size expressed as IS/AAR. In addition, fibrosis was quantified at the end of the experiment by randomly selecting three heart samples from each group and staining longitudinal 6 *μ*m heart sections with Masson trichrome histochemistry. For each heart section, 5 nonoverlapping areas of the LV immediately proximal to, but not including, the coronary ligation site were imaged using an Olympus BX51 optical microscope (Center Valley, PA, USA). Using ImageJ software, areas of fibrosis within all images were measured. The analysis consisted of determining the MT stained areas (blue) and nonstained myocyte areas from each section via color-based thresholding. A final fibrosis score per heart was achieved by averaging the percentage of total fibrotic areas, calculated as the (total area of collagen/total area of image) × 100%.

### 2.7. Investigation of Molecular Mechanisms of Action by Western Blot Analysis

The frozen ischemic myocardium was ground to a fine powder using mortar and pestle and the liquid nitrogen was allowed to evaporate. Then, the frozen tissues were homogenized in 500 *μ*L of ice cold lysis buffer (0.5 M Tris-HCl, 150 mM NaCl, 0.1% SDS, 1 mM EDTA, 1% NP-40, 1 mM NaF, 1 mM Na_3_VO_4_, 1 mM PMSF, and 1 mM aprotinin, containing protease inhibitors) in a glass-Teflon tissue grinder. After homogenization, the samples were centrifuged at 12,000 rpm for 10 min at 4°C and the supernatants were collected. The amount of protein was measured using the BCA assay. For Western blot analysis, the samples (containing 30 *μ*g of total protein) were added with the same amount of the sample loading buffer (reducing buffer) and then denatured at 100°C with boiling water for 5 min. Protein was then separated by electrophoresis on a 10% sodium dodecyl sulfate-polyacrylamide gel and transferred to a polyvinylidene fluoride membrane (Amersham Biosciences, Buckinghamshire, United Kingdom). The membrane was blocked in 5% skim milk in Tris-buffered saline containing 0.1% Tween-20 (TBS-T) and incubated with primary antibodies overnight at 4°C. The primary antibodies were HSP20 (sc-51955, Santa Cruz Biotechnology Inc.), HSP27 (sc-1049, Santa Cruz Biotechnology Inc.), and HSP70 (sc-1060, Santa Cruz Biotechnology Inc.). The membranes were washed and subsequently incubated with dilutions of secondary antibodies, and the membranes were detected using an enhanced chemiluminescence system (GE Healthcare). The relative intensity of each protein band was normalized to the intensity of GADPH.

### 2.8. Statistical Analysis

All values are presented as the mean ± standard error of the mean. A one-way analysis of variance followed by the Student-Newman-Keuls multiple comparison test was used to analyze group differences in single point data regarding infarct size. *p* values less than 0.05 were considered statistically significant. Statistical analyses were performed using SPSS for Windows, version 12.0 (SPSS Inc., Chicago, IL).

## 3. Results

### 3.1. Infarct Size/Area at Risk and Myocardial Fibrosis

**Figure 3:**
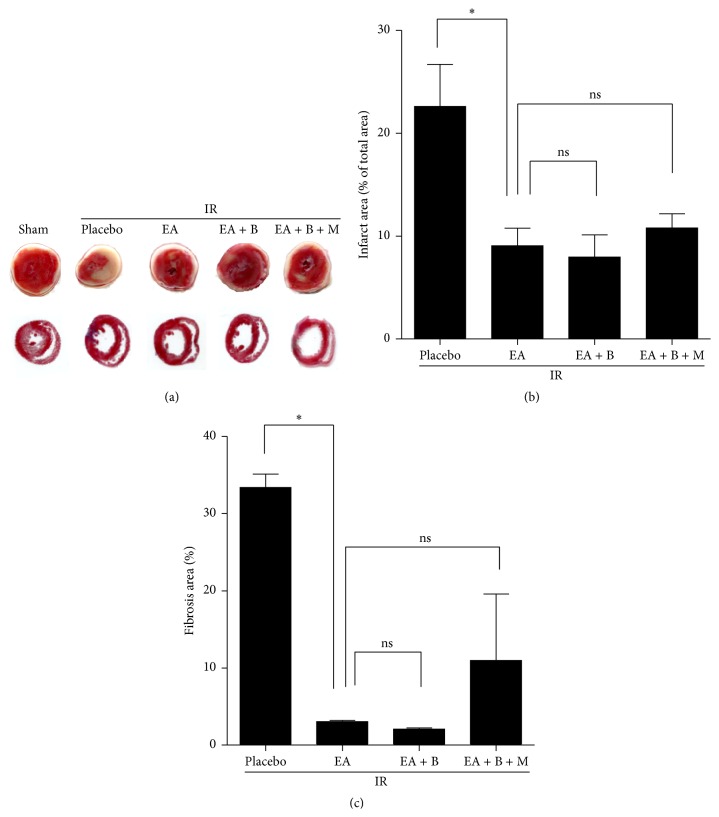
Infarct size and fibrosis area. (a) Representative TTC staining and MTC staining heart slices of LV area. (b) Infarct size expressed as a percentage of the area at risk in rats. ^*∗*^ *p* < 0.05 versus the placebo group. (c) Fibrotic area of selected points expressed as a percentage of the total area. ^*∗*^ *p* < 0.05 versus the placebo group. EA, electroacupuncture group; EA + B, electroacupuncture + back-shu electroacupuncture group; and EA + B + M, electroacupuncture + back-shu electroacupuncture + moxibustion group.

The infarct size within the percentage of the risk zone was 2.20 ± 2.40% and 23.15 ± 3.71% in the control group and placebo control group, respectively, indicating that the animal model was well established. Myocardial injury was diminished in the EA, EA + B and EA + B + M groups, as seen from the lower infarct area relative to the total area (EA, 8.90 ± 7.13%; EA + B, 7.93 ± 2.95%; EA + B + M, 10.89 ± 2.40%; *p* < 0.05). However, the decrease in infarct size of the EA + B group and the EA + B + M group was not statistically significant compared to the EA group (EA versus EA + B: 8.90 ± 7.1 3% versus 7.93 ± 2.95% and EA versus EA + B + M: 8.90 ± 7.1 3% versus 10.89 ± 2.40%; *p* > 0.05) (Figure 3).

No myocardial fibrosis was observed in the control group, and extensive fibrosis was observed in the placebo group (33.33 ± 3.38%). Mild fibrosis was observed in the EA group (3.03 ± 0.33%), and the EA + B group had less severe fibrosis than the EA group (2.03 ± 0.42%). However, the EA + B + M group had a larger fibrotic area than the other treatment groups (11.73 ± 17.23%). The degree of attenuation of infarct size was not statistically significant between the three treatment groups (EA versus EA + B: 3.03 ± 0.33% versus 2.03 ± 0.42%, *p* > 0.05).

### 3.2. Cardiac Function

**Figure 4:**
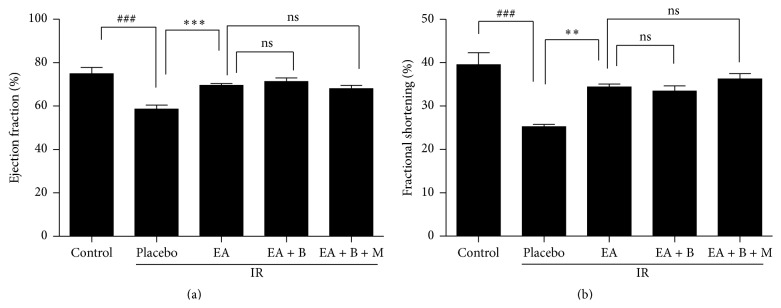
Results of 2D echocardiography. (a) Ejection fraction and (b) fractional shortening of the five groups. Both ejection fraction and fractional shortening were significantly different between the placebo group and the three treatment groups. EA, electroacupuncture group; EA + B, electroacupuncture + back-shu electroacupuncture group; and EA + B + M, electroacupuncture + back-shu electroacupuncture + moxibustion group. ^###^ *p* < 0.05 versus the control group and ^*∗∗∗*^ *p* < 0.05 versus the placebo group. ^*∗∗*^ *p* < 0.01 versus the placebo group.

Echocardiography revealed dilated left ventricle and reduced cardiac performance in animals with IR injury. Both EF and FS were improved by the three different treatment modalities compared with placebo treatment (Table 2, Figure 4). However, there were no significant differences between the three treatment groups.

### 3.3. Production of Heat Shock Proteins in the Heart from Rats Subjected to Ischemia and Reperfusion

**Figure 5:**
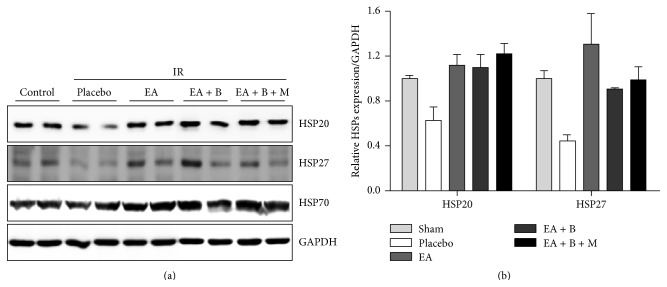
HSP20, HSP27, and HSP70 expression. (a) The production of HSP20, HSP27, and HSP70 as detected by Western blotting. (b) Overexpression of HSP20 and HSP27 in the three treatment groups compared to placebo group. EA, electroacupuncture group; EA + B, electroacupuncture group + back-shu electroacupuncture group; EA + B + M, electroacupuncture group + back-shu + moxibustion group.

Western blotting was used to determine the myocardial content of HSP20, HSP27, and HSP70.  Figure 5 shows the major peptide band at 65 kDa, representing the specific isoform of each heat shock protein in different groups. We observed significant overexpression of HSP20 in the three treatment groups compared to the placebo group (*p* < 0.05), significant overexpression of HSP27 in the EA group compared to the placebo group (*p* < 0.05), and significant overexpression of HSP70 in the EA + B + M group compared to the placebo group (*p* < 0.05). However, there were no significant differences between the three treatment groups for HSP20, HSP27, and HSP70.

## 4. Discussion

We investigated possible synergistic effects of three treatment methods (EA, back-shu EA, and moxibustion) that work through different pathways and analyzed the results. Previous studies in our lab suggested that adding an identical treatment from one to both sides of the body, increasing the number of distal acupuncture points, and increasing the number of treatment sessions in a trial do not strengthen preconditioning effects [not yet published]. This study investigated whether increasing treatment does not lead to increased effects even if the additional treatments worked through different mechanisms. Our results showed that the cardioprotective effects of all three treatment groups were significant compared to the placebo group; however, additional treatment on back-shu points and moxibustion did not significantly decrease infarct size.

We chose these three treatment methods based on literature review and clinical experience. PC6 and ST36 are two of the most frequently investigated acupuncture points for treating cardiovascular problems in the laboratory and in the clinic [21]. PC5 and ST37 were additionally needled in order to conduct EA stimulation on PC6 and ST37, and many cardiovascular studies have used these pairs to produce cardiovascular protective actions [22]. These points send strong signals to the central nervous system via types III and IV sensory nerve fibers on the median and deep peroneal nerves [12]. Suggested mechanisms include the release of endogenous opioids in the brain stem (which leads to a decrease in sympathetic outflow and norepinephrine release in the ischemic area [21]) and the involvement of beta-adrenoceptors [23]. A recent study also showed that acupuncture at PC6 and ST36 improved ECG findings in rats with acute cardiac ischemia by increasing c-fos expression in the nucleus of the solitary tract, which is a center that integrates cardiac functional activity [17].

Back-shu points and moxibustion are hypothesized to exert effects quite differently from PC6 or ST36. Stimulation of back-shu points regulates the autonomic nervous system [24], since they are located above the T4 and T5 sympathetic nerves, close to the dorsal ganglia and the sympathetic innervations to the heart. Studies that looked into the effects of back-shu points for other diseases such as asthma also showed that back-shu points can directly stimulate the dorsal root ganglia to produce substance P [13]. As for moxibustion or LSTS, a recent proteomic analysis study concluded that EA and LSTS specifically act through different myocardial protective mechanisms [25, 26]. However, we decided to add back-shu points first because using a single treatment modality would make it easier to clarify the mechanisms later on. We added moxibustion as a preliminary investigation of whether the addition of another treatment would increase the effects even further.

The treatment protocol of this trial was intended to be clinically relevant, which is why we chose postconditioning methods over preconditioning. Preconditioning treatments are limited to situations where the onset of ischemia can be predicted, such as planned cardiovascular surgeries. Since most incidences of myocardial infarctions are unpredictable, we focused on conditioning the rats just before reperfusion, which is usually the case in emergency coronary artery bypass surgeries or percutaneous coronary interventions. Measurement of the infarct size 5 days after ischemia provided data on early- and late-phase effects and acute and collective acupuncture effects. In the real world, patients are likely to receive acupuncture treatment more than once and it is necessary to look into the long-term and cumulative effects of these treatments.

There are a few limitations to our study. One limitation that applies to most animal studies related to this subject is the fact that these trials are conducted on healthy and young animals. Past studies have shown that preconditioning or postconditioning effects are different between young and old rats [27]. Since the long-term focus of our trial was to design a treatment package suitable for future clinical trials, it would have been more practical to look into additional protective effects in older rats. This is important because previous trials that have investigated age and sex have also shown that the preconditioning effects were more evident in middle-aged rats compared to younger ones [28, 29].

As for the increase in infarct size after additional moxibustion treatment, we believe that either the moxibustion stick that we used had different qualities from the heat generator placed 0.5 cm above PC6, which is more frequently used in LSTS, or more treatment does not always produce better results. This is in agreement with a few other studies that have attempted to increase treatment effects by increasing the stimulation period. In rat ischemic brain injury, 45 min of EA treatment did not lead to as much improvement as 30 min of EA stimulation [15]. In addition, in human experimental cold thermal pain models, 30 min of EA stimulation resulted in better results than 40 min of stimulation [30]. Myocardial protection with postconditioning was not enhanced by ischemic preconditioning [31].

We postulated that EA on PC6, ST36, BL14, and BL15 and moxibustion on PC6 act through different pathways, but all of these treatments are, in a large sense, external stimulations of the body that aim to activate self-protective mechanisms. Physiologically, the mechanism through which acupuncture is known to work still falls within the category of mechanical stimulation on afferent nerve fibers to elicit local, segmental, or systemic effects. Even if different acupuncture points were utilized, all neural pathways could have reached maximal treatment effects. However, since this was a preliminary investigation, it is necessary to continue to explore conditioning methods that may increase overall effects on the myocardium. This may include investigating acupuncture treatment with other pharmaceuticals, since oral administration of geranylgeranylacetone (GGA) and LSTS doubled the induction of heat shock protein 70 (HSP 70) in rat livers [32]. It is also imperative to carry on related studies onto the bedside, which has already begun [33, 34]. Our trial was an initial attempt to explore potential therapeutic improvements in order to provide better treatment strategies for clinical trials.

## Figures and Tables

**Table 1:** Acupuncture treatment according to STRASA^*∗*^.

Intervention	Item	Description
Experimental animals	(a) Animal model	Nonpain model
	(b) Details for animals	Sprague-Dawley rats of 8 to 9 weeks of age, weighing 250–300 g each
	(c) Experimental environment	Room temperature 24 ± 1°C, room humidity 63 ± 5%

Type of acupuncture	(a) Statement about the type of acupuncture used	Electroacupuncture (ES-160, ITO, Tokyo, Japan)
	(b) Rationale for treatment	Literature review and previous studies

List of points	(a) Name of points	PC5, PC6, ST36, ST37, BL14, BL15
	(b) Literature sources to justify rationale	Previous literature and animal studies (listed among references)
	(c) Location of point	(i) PC5: approximately 2-3 cm proximal to PC6, just above the median nerve (ii) PC6: inside the arm, approximately 4 cm proximal to the base of the palmar wrist crease, just above the median nerve (iii) ST36: on the anterior aspect of the knee, approximately 9 cm below the depression point lateral to the patellar ligament and 5 mm lateral to the anterior tubercle of the tibia (iv) ST37: approximately 9 cm below (distal) ST36 (v) BL14: on the outside dorsal spine corresponding to the 4th thoracic vertebrae (vi) BL15: on the outside dorsal spine corresponding to the 5th thoracic vertebrae

Needle stimulation	(a) Depth of insertion	PC5, PC6: 5 mm ST36, ST37, BL14, BL15: 1 cm
	(b) Needle stimulation technique	Electroacupuncture (2 Hz)
	(c) Needle retention time	30 min
	(d) Specification of needle	0.20 × 30 mm, Dongbang Acupuncture Inc., Gyeonggi-Do, Korea
	(e) Responses elicited	Motor threshold response (movement of the paw and visible muscle twitches of the stimulated leg)

Treatment regimen	(a) Number of treatment sessions	Five sessions
	(b) Frequency of treatment	Once per day

Cointervention	Other interventions	None

Practitioner background	(a) Duration of relevant training	Six years
	(b) Length of clinical experience	Four years
	(c) Expertise in specific condition	None

Control interventions	(a) Active comparison point	Away from acupuncture points and away from near meridians, at a shallower depth of insertion
	(b) Control stimulation	Manual acupuncture
	(c) Details of control intervention	Control points for PC5, PC6: two points located between the Triple Energizer meridian and the Small Intestine meridian Control points for ST36, ST37: two points located between the Gall Bladder meridian and the Bladder meridian
	(d) Sources that justify choice of control	Previous studies (the selection of acupuncture points was based on the results of previous studies)

^*∗*^Standards for Reporting in Animal Studies of Acupuncture (STRASA) guidelines; PC, pericardium; ST, stomach; BL, bladder.

**Table 2:** Results of transthoracic echocardiography.

Group	*n*	LVEF (%)	LVFS (%)	LVIDd (mm)	LVIDs (mm)	EDV (mL)	ESV (mL)
Control	7	74.91 ± 8.30	39.56 ± 7.80	6.77 ± 0.65	4.13 ± 0.85	0.72 ± 0.18	0.19 ± 0.09
Placebo	7	56.73 ± 3.89^#^	25.98 ± 2.41^#^	7.32 ± 0.84	5.42 ± 0.66	0.73 ± 0.34	0.39 ± 0.13
EA	7	69.03 ± 3.68^*∗*^	34.23 ± 2.83^*∗*^	7.05 ± 0.76	4.64 ± 0.55	0.81 ± 0.24	0.25 ± 0.08
EA + B	7	67.87 ± 4.19^*∗*^	33.49 ± 3.03^*∗*^	7.57 ± 0.79	5.03 ± 0.56	0.98 ± 0.28	0.31 ± 0.09
EA + B + M	7	67.72 ± 11.21^*∗*^	33.93 ± 7.26^*∗*^	7.65 ± 0.43	5.05 ± 0.62	1.00 ± 0.16	0.32 ± 0.12

Values are mean ± SE. EA, electroacupuncture group; EA + B, electroacupuncture + back-shu electroacupuncture group; EA + B + M, electroacupuncture + back-shu electroacupuncture + moxibustion group. *n*, number of rats in each group; LVEF, left ventricular ejection fraction; LVFS, left ventricular fractional shortening; LVIDd, left ventricular internal diameters in diastole; LVIDs, left ventricular internal diameters in systole; EDV, end diastolic volume; ESV, end systolic volume. ^#^ *p* < 0.05 versus the control group. ^*∗*^ *p* < 0.05 versus the placebo group.
